# Towards combinatorial mixing devices without any pumps by open-capillary channels: fundamentals and applications

**DOI:** 10.1038/srep10263

**Published:** 2015-06-23

**Authors:** Marie Tani, Ryuji Kawano, Koki Kamiya, Ko Okumura

**Affiliations:** 1 Department of Physics, Faculty of Science, Ochanomizu University, 2-1-1, Otsuka, Bunkyo-ku, Tokyo 112-8610, Japan; 2 Division of Biotechnology and Life Science, Tokyo University of Agriculture and Technology, 2-24-16 Naka-cho Koganei-shi Tokyo 184-8588 Japan; 3 Artificial Cell Membrane Systems Group, Kanagawa Academy of Science and Technology (KAST), 3-2-1 Sakado Takatsu-ku Kawasaki 213-0012, Japan

## Abstract

In chemistry, biology, medical sciences and pharmaceutical industries, many reactions have to be checked by transporting and mixing expensive liquids. For such purposes, microfluidics systems consisting of closed channels with external pumps have been useful. However, the usage has been limited because of high fabrication cost and need for a fixed setup. Here, we show that open-capillary channels, which can be fabricated outside a clean room on durable substrates and are washable and reusable, are considerably promising for micro-devices that function without pumps, as a result of detailed studies on the imbibition of open micro-channels. We find that the statics and dynamics of the imbibition follow simple scaling laws in a wide and practical range; although a precursor film obeying a universal dynamics appears in the vertical imbibition, it disappears in the horizontal mode to make the design of complex micro-channel geometry feasible. We fabricate micro open-channel devices without any pumps to express the green florescent protein (GFP) by transporting highly viscous solutions and to accomplish simultaneous chemical reactions for the Bromothymol blue (BTB) solution. We envision that open-capillary devices will become a simple and low-cost option to achieve microfluidic devices that are usable in small clinics and field studies.

Micropatterned surfaces are ubiquitous in nature and found, for example, on the surface of plants^1^, insects^2^,^3^, and animals^4^,^5^. Partly because of the inspiration from such natural structures, extensive studies on micropatterned surfaces^6^have been devoted for understanding and controlling specific wetting properties such as superhydrophobicity^7^and leophobicity^8^. Accordingly, such textured surfaces have allowed various applications, which include microfluidics devices^9^,^10^such as liquid-drop transport^11^,^12^,^13^, controlled and patterned film coating^14^,^15^, and slippery pre-suffused surfaces^16^,^17^. In particular, combinatorial mixing of small amounts of liquids^18^,^19^,^20^is an important issue in chemistry, biology, medical sciences and pharmaceutical industries^10^,^21^,^22^,^23^. Note that liquids are usually expensive, as in the case of the DNA microarray^24^,^25^,^26^,^27^and the crystallization of proteins^28^,^29^.

One of the keys for these applications is the knowledge of the imbibition dynamics for such patterned surfaces and, in particular, the availability of simple scaling laws for the dynamics should be insightful and useful^30^,^31^. The dynamics has actively been studied for model surfaces on which an array of micro posts are arranged (see, for example, Ref. 32). Quite frequently, the imbibition length 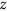 scales with the square root of elapsed time 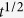
14,33,34, as in the viscous regime of the capillary rise^30^,^35^, but with the coefficient dependent on the geometry of posts (pillar height, distance, and radius). In fact, two simple scaling regimes for the coefficient have been identified^33^depending on the ratio of the pillar height to the pillar distance. The different dynamics *z*  ~  *t*^1/3^ has also been found in different situations^36^,^37^,^38^, together with other slowing-down dynamics observed for imbibition of papers^39^,^40^and an unusual linear dynamics found for imbibition on the surface of legs of a certain animal^5^.

In this study, we investigate the imbibition dynamics for a simple open-capillary channel whose section is rectangular, which is fabricated by a micro-milling machine outside a clean room with no need for delicate and fragile adhesion process. It is emphasized here that the capillary rise into open capillaries is not a resolved problem but an important current problem (see Fig. 1). As detailed in Discussion, this type of capillary rise, which includes the imbibition of textured surfaces as well as rectangular open channels, is associated with the growth of a large free liquid-air interface as a result of the imbibition, and this feature introduces highly nontrivial issues. In fact, the shape of the large free liquid-air interface seems to be a key factor for determining the dynamics: different scaling laws have recently been established for various open capillary channels, which include the dynamic expressed as *z*  ~  *t*^1/3^ (see Fig. 1a–c and Refs. 37,38), *z*  ~  *t*^1/2^ (see Fig. 1d and e, and Refs. 14,33,34,41), and 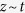 (see Fig. 1f and Ref. 5). Since such scaling laws are highly useful for the development of the device for the transport of a small amount of liquid, studies on the imbibition of open capillary is indispensable not only from a fundamental viewpoint but also from a practical viewpoint. If we consider that the recent high activities in the study of the imbibition of open capillaries of various types, some of which are illustrated in Fig. 1, it is highly expected that new scaling regimes will be found from the imbibition of rectangular open channels.

In the present study on rectangular open channels, we find that a liquid column filling the channel stops at a certain height as in the conventional capillary rise. Remarkably, a simple approximate theory in the deep-channel limit turns out to be extremely robust: the scaling laws derived for the rising dynamics in the deep-channel limit can correctly describe the dynamics observed even in rather shallow channels. In addition, we find that a precursor film develops gradually and continues to rise even after the liquid column stops rising. The dynamics of the precursor film is revealed to be universal, i.e., independent of channel geometry, and explained basically by the theory discussed in previous studies^36^,^37^,^38^. This film, which potentially limits practical applications, is, however, suppressed when the imbibition proceeds in the horizontal direction. Together with this disappearance of the precursor film, the simple quantitative laws for the final height and the dynamics established for the liquid column allow the design of complex micro-devices for transporting small amounts of liquids without any flow systems, such as electronic pumps. We provide simple guiding principles for developing such devices on the basis of the simple laws.

To show the capability of rectangular open channels for practical applications, we develop microfluidic mixing devices that function without any pumps. As an example, we demonstrate simultaneous multiple color changes of BTB solution. We further demonstrate a cell-free protein synthesis^42^that expresses GFP^43^, which is much simpler than previous devices for the *in vitro* protein synthesis^44^that require sealed and molded channels developed with photolithography with a flow system. As a result of quantitative analysis of the demonstrations, we show that possible drawbacks of open-channels, such as contamination and evaporation, are not a significant problem in typical chemical and biochemical reactions. In many cases, rectangular open channels function equally well compared with closed ones, even though rectangular open channels are much more easy to make, durable, and much less expensive.

## Results

To study the capillary rise on an open capillary, we fabricated rectangular open channels of length around several centimeters on a hard transparent PMMA plate. The section of the channel is rectangular of width 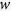 and depth 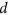. The width 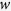 is about 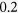 mm while 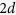 is set to be larger than 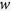 to make theoretical prediction easier as we see below.

To observe imbibition, we made one edge of the plate in contact with a viscous liquid, the polydimethylsiloxane (PDMS) solution (commonly known as silicone oil), with keeping the plate in the upright position, perpendicular to the liquid’s horizontal surface, and quantified the dynamics of the capillary rise from snapshots; the combination of the oil with PMMA substrates allows the complete wetting, making the contact angle zero. A typical time evolution of the rising dynamics is demonstrated in a series of snapshots in Fig. 2a (1). At later stages, two different fronts of the liquid become distinguishable. This is because a thin precursor film (PF) develops gradually ahead of the front of the bulky liquid column (LC), below which the rectangular channel is almost completely filled. The positions of the two fronts, one for the precursor film and the other for the liquid column, are plotted as a function of elapsed time in Fig. 2a (2). While the length of the liquid column saturates to a fixed value as in the usual capillary rise, the precursor film keeps proceeding to the top of the plate. For further details on experiment, see Methods.

### Statics and dynamics of the liquid column

As shown in Fig. 2b (1), the final saturated length of the liquid column seems independent of kinematic viscosity 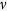 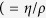, with 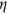 the viscosity and 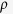
the density), and dependent on the channel depth 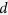 only weakly. To predict this final height 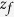 of the column, we assume that the section of the column is rectangular of area 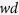 and that the liquid completely wets the sides and bottom of the channel. As a result of energy minimization, we obtain in Methods
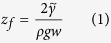
where 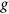 is the gravitational acceleration and 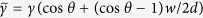, with 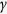 the surface tension of the liquid and 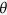 the contact angle. In the present case of complete wetting, the value of 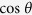 is practically equal to one.

Remarkably, this formula reproduces the experimental observations quite well, in the range of parameters varied in the present study: the section of the liquid column is practically rectangle. Because of this, we can determine a good estimate of the average value of 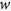 from this height measurement. The weak dependence on 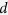 of 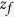 mentioned above actually comes from slight differences in 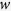 (values of 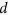 in the plot are not the exact ones, as indicated in Methods).

In fact, the free surface of the liquid column filling open channels becomes concave with height to guarantee the pressure drop, although the rectangular approximation works well. As discussed in Methods, the radius of the curvature simply scales as 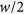 at the front of the liquid column^41^; the curvature of the free surface of the liquid column increases with the height, from zero (flat surface) at the bottom to 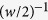 at the top.

The initial regime of the dynamics can be described by the competition between capillary drive and viscous drag. We consider the limit in which the viscous dissipation associated with the sides of a channel dominates over that associated with the bottom of the channel, i.e.,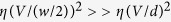, or equivalently, 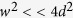, which is fairly well satisfied in the present study. In this limit, the initial dynamics is derived in Methods as
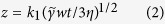
with 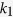 a numerical constant. This constant is close to one when the infinite-plate approximation is good (see Methods), e.g., when 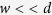. By using 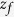 given in Eq. (1), a dimensionless form of the dynamic law is given by
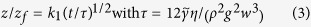


The final regime of the dynamics can be described by the competition of capillarity, gravity, and viscosity. In the same approximations for the initial dynamics, the final dynamics is derived in Methods as
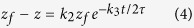
with 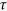 given above, 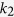 an undeterminable integration constant, and 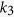 a constant close to one for 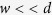.

As demonstrated in Fig. 2b (2), the initial regime is well described by Eq. (2) or (3) at a quantitative level, with 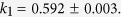 When we collect all the data in Fig. 2b (1) and plot the data with the renormalized axes specified by the theory, all the data collapse remarkably well onto the predicted master curve; The condition for the approximation is 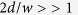, while the values of 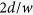 are 10, 4, and 2 for 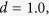 0.4, and 0.2 mm, respectively: the scaling law obtained in the deep-channel limit is valid even for rather shallow channels. The initial regime is practically valid when 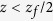 (half of the final height) as seen from Fig. 2b (2).

The final regime is well captured by Eq. (4) with 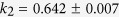 and 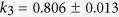 as demonstrated in Fig. 2b (3), in which the vertical and horizontal axes are arranged so that all the data collapse onto a single straight line in the regime if the theory works well. As seen in the main plot of Fig. 2b (3), the data (except for 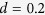 mm, for which the condition for the approximate theory is only marginally satisfied) collapse onto a single master line, with the inset showing slight deviation of the data at 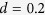 mm.

### Dynamics of the precursor film

As indicated above, the precursor film does not seem to stop (as long as the evaporation of the film is negligible), which reminds the capillary rise into corners^36^,^37^and into textured surfaces decorated with short and rounded pillars^38^. It is known that in such cases the imbibition becomes universal in a sense that the dynamics does not depend on the geometry of channels. The universal scaling form for the imbibition length 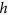 in the present case is given as
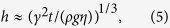


where the complete wetting is assumed. This is derived in Refs. 36, 37 but the derivation at the level of scaling laws is given in Methods for completeness. Note here that as seen from the derivation the existence of the precursor film can be suppressed for small 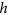 and small effective gravity (e.g. in the horizontal mode experiment). This feature is preserved irrespective of geometrical parameters of the channel such as 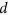 and 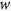, i.e., universal in the previous sense. In the dimensionless form, this relation is given as
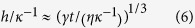
with 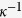 the capillary length defined as 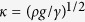.

As demonstrated in Fig. 2c, we see clearly that the length of precursor film 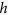 as a function of time approaches the scaling predicted in Eq. (5) or (6) for 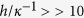. This limited agreement is fully consistent with the prediction, which holds only in the limit when *h* 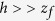, i.e., 
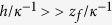
 with 
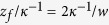
 being in the range from 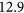 to 13.5. Note that if channels were too long, the problem of evaporation would come into play.

### Microdevices for mixing solutions

To demonstrate the capability of open channels for micro mixing devices, we designed a simple device for mixing one solution with four different solutions and fabricated it on a PMMA solid plate (see Fig. 3a (1) and Fig. S1). We first deposit four solutions at the rectangular spots, which are pinned by the edges of the spots. We then inject another solution in the circular spot. Soon after the injection, the solution is transported by capillary force to the four rectangular spots. Demonstration in Fig. 3a (1) was performed by using the BTB solution and four solutions with different pH. It is emphasized here that we do not need to seal the system with a top plate. This is advantageous in terms of fabrication, because the sealing process with adhesive is technically difficult for PMMA. The quantitative analysis in Fig. 3a (2) shows that the mixing at the four spots (S1 to S4) start to occur almost simultaneously.

We further fabricated a simpler device for mixing two solutions (see Fig. S2); the device consists of two circular spots S_1_ and S_2_, with each connected by open channels (C_1_ and C_2_, respectively) to a rectangular spot M for mixing. We deposit two solutions, one at S_1_ and the other at S_2_; as soon as the deposition is finished, the solutions start to proceed and reach the mixing spot “simultaneously” (see Discussion for the details) to initiate certain reactions. As demonstrated in Fig. 3b (1), we successfully synthesized a protein with the device. To quantify the result of mixing, the brightness is shown as a function of time in Fig. 3b (2).

This demonstration of the GFP expression highlights that this device is practically useful for biochemical reactions in general, as suggested by the following three remarks. (1) The mixing power of device without using any mechanical or electronic pumps is strong enough for highly viscous liquids. Rough estimates of viscosities of the two solutions mixed in the demonstration are approximately 1 and 10 mPa
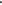
s. (2) The evaporation from the free liquid interface is not significant for the duration approximately 40 min. after the start of the reaction, as indicated in Fig. 3b (the rightmost symbol for each reaction is filled in Fig. 3b (2) to show that the evaporation becomes significant at the final stage as in the bottom snapshot in Fig. 3b (1)). (3) The reaction time for biochemical reactions can be estimated by this device: the plots of the mean brightness show a plateau (see Discussion for the details) in a reproducible way (Fig. 3b (2)), from which the reaction time is estimated as about 30 min. In fact, this estimate is a reasonable one, considering other reported values in the literature (e.g., Ref. 45).

We could not observe any effects of the precursor film in the two demonstrations, in which the devices were placed horizontal, as explained in Methods: the precursor film tends to disappear in the small gravity limit, i.e., when the devices are placed horizontal.

## Discussion

Our study resolves the following problems that have limited practical application of the previous studies on mixing solutions by capillary force^46^: (1) Only conventional closed micro-channels have been discussed for mixing, which are less attractive than open channels because of various advantages of open channels as discussed in the next paragraph and because of the readiness for use with pumps at the cost of expensive facilities for fabrication. (2) Control of the flow has seemed difficult for capillary channels because no simple scaling laws clarifying intuitive physical pictures have been exploited for the design of mixing system. Detailed analyses have been available for closed channels^47^, open V-shaped channels^48^, and also for open rectangular channels^49^,^50^. In the most recent article^50^, for example, the authors derived an approximate formula corresponding to Eq. (2) but they did not discuss possible scaling regimes for the geometry of channels including ours (the deep channel limit). In addition, their experiments are performed in a parameter region away from the deep channel limit. On the other hand, we derived a significantly simplified version of their formula via much simpler physical arguments and experimentally showed that the simplified version is nearly exact for a wide parameter range useful for practical application. In this sense, the scaling laws in Eq. (2) [and also Eqs. (1) and (4)] are established for the first time in this study (On the contrary the scaling law in Eq. (5) itself has already been confirmed previously but is here confirmed in a completely new context). This is fundamentally important for the development of devices, since simple scaling laws are much easier to handle and much more useful than detailed formula (unnecessary much more complex in the parameter range of interest) in developing practical devices. (3) No demonstration has been available that shows the advantage of capillary mixing over pump-driven mixing especially when mixing viscous liquids, which are important in biological or medical applications (e.g., the viscosity of blood is several times as high as water), although such demonstrations are indispensable as a bridge between practical applications and simple physical understandings. These three difficulties are overcome in this study: we show that simple scaling laws hold for the imbibition of rectangular open-capillary channels in a wide range on the basis of clear physical pictures; the scaling laws make the design of complex mixing device simple for rectangular open channels; we demonstrate an example of mixing viscous bio-solutions by expressing GFP and a simple example of combinatorial mixing by using BTB.

Open-channel devices are advantageous in the following respects if proper care is taken for evaporation and contamination due to direct contact of liquid with air: (1) Less demanding fabrication process. Open-channel devices are fabricated with an automatic milling machine outside a clean room on PMMA and sealing by another transparent plate, which is difficult for PMMA, is unnecessary. (2) Durability for reuse and washing. PMMA is mechanically much stronger than PDMS gels conventionally used for closed-channel devices. In addition, open-channel devices are free from adhesion for lids (since there are no lids to seal the devices) and from connection to a pump system, both of which usually undermine durability of closed-channel devices. In fact, Movie S1 on the demonstration for the BTB solution proves a possibility of reuse: the experiment recorded on the movie is an experiment performed after many times of the same experiment on the same device with washing (supersonic washing or simple rinsing by pure water) and/or surface treatment being performed between experiments. (3) Advanced control of surface wettability. Without a sealing plate and with high durability, wetting properties of the inside surface of the path are controlled effectively and repeatedly by a variety of surface treatments. For example, in the BTB demonstration, an advanced control is performed (see Methods). (4) Collection of liquids after reactions. The absence of lids gives flexibility for the collection. Considering various advantages of open-capillary mixing devices as discussed above, we envision that fundamentals and applications demonstrated here will open a way to the design of less expensive portable microdevices for combinatorial mixing of small amounts of liquids in the near future for various purposes such as complex medical tests at a small clinic.

The precursor film discussed here is completely different from that discussed in the framework of the Tanner’s law^30^. Several remarks follow on this. (1) The precursor film should always exist in the sense of Tanner’s law in principle and the existence has nothing to do with gravity. However, the Tanner’s precursor film is not observed in our case because of the limitation of our experimental setup: such precursor films are in general difficult to observe when a macroscopic observation is made as in the present experiment. (2) We rather claim here that our “ macroscopic” precursor film ahead of the growing liquid column in the rectangular channel in the upright position originates from the phenomenon of capillary rise into corners, well established in Refs. 36, 38, whereas the derivations in Theoretical Methods for the capillary rise into corners give a simple and insightful interpretation at the level of scaling laws. (3) Our interpretation that the “macroscopic” precursor film is the film advancing into the corners is justified in Fig. 2c (2): the data approach a straight line with the slope 1/3, as predicted by the theory for the capillary rise into corners. (4) The disappearance of the “ macroscopic” precursor film or capillary rise into corners in the horizontal mode is in accordance with the fact that the basic assumption of the theory of capillary rise into corners is no longer valid in the horizontal mode, i.e., under no gravity (see Theoretical Method). (5) The Tanner’s precursor film can be negligible in practical applications, because the imbibition speed of liquid column is significantly quick compared with the development of the Tanner’s precursor film, and, in fact, in the demonstrations we find that mixing starts at the moment the front of the liquid column arrived at the mixing spots; otherwise, the mixing would start before the arrival of the column front.

As for the analysis on the concentration of the expression of GFP, the thickness of liquid film in the mixing spot during the reaction changes with time because of evaporation as inferred from Fig. 3b (1) and Movie S2. However, the depth of focus of the lens is only 10 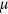
m, which is much smaller than the thickness of the liquid film occupying the mixing spot (this thickness is 600 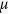
m). This might be the reason we could observe the plateau before evaporation in Fig. 3b (2).

Practically, it is important to design the mixing device considering the order of the reaction time. Liquids were injected to Spots S_1_ and S_2_ simultaneously in the GFP case. We used a pipette with two tips (outlets) the distance of which is equal to that of Spot S_1_ and S_2_. A single plunger of the pipet allows simultaneous injection of the liquids in the two spot. However, in the GFP demonstration, the simultaneousness is much less severe than in the BTB demonstration. This is because the reaction time is much longer in the GFP case. Considering this we employed the simple design for the device shown in Fig. S2 (the distance from Spot S_1_ to M is equal to that from Spot S_2_ to M).

**Figure 1:**
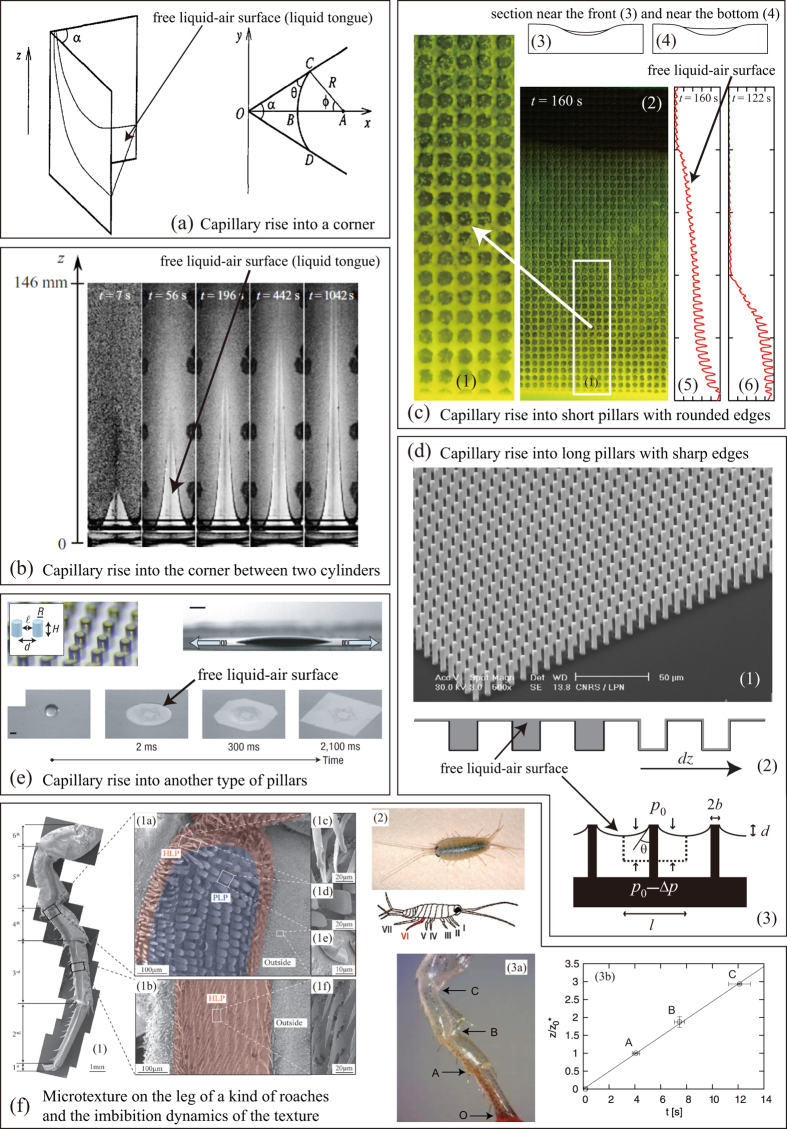
Various types of the open-capillary rise accompanied by a large free liquid-air surface that develops with the imbibition. The shape of free surface is determined dynamically and the imbibition dynamics changes depending on the shape of the free surface. This fact suggests that the capillary rise into rectangular open capillaries is a nontrivial current issue. (**a**) Capillary rise into a corner: the imbibition height 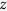 scales as *z*  ~  *t*^1/3^. Reprinted from Ref. 36 published in 1994. (**b**) Capillary rise into the corner created between two cylinders (the cylinders are aligned vertically so that the cylinders are in contact with each other, whereas the bottom circles are touched with the liquid for imbibition). The imbibition dynamics is again described by *z*  ~  *t*^1/3^. Reprinted from Ref. 37 published in 2011. (**c**) Capillary rise into short pillars with rounded edges. Remarkably, the dynamics is still expressed as *z*  ~  *t*^1/3^. As indicated in the figures the liquid film thickness thins down towards the film front. Reprinted from Ref. 38 published in 2012. (**d**) Capillary rise into long pillars with sharp edges. This dynamics is expressed as *z*  ~  *t*^1/2^. In contrast with c, the liquid film thickness is comparable with the pillar height, as indicated in d (2). As indicated in d (3), the sharp edge of a pillar pins the free liquid-air interface, which deforms the interface to create pressure drop that drives the imbibition. Illustration d (1) and d (2) are reprinted from Ref. 33 published in 2007, and illustration d (3) is from Ref. 41 published in 2009. (**e**) Capillary rise into another type of pillars. This dynamics is still expressed as *z*  ~  *t*^1/2^ (a similar dynamics is also found in Ref. 34). As in d, the liquid film thickness is comparable to the pillar height, as indicated by the bottom 4 photographs. Reprinted from Ref. 14 published in 2007. (**f**) Microstructure found on the leg of a small animal shown in f (1)-(2) and the linear imbibition dynamics *z*  ~  *t* (in f (3)). Illustration f (1) and f (2) are reprinted from Ref. 57 published in 2013 and illustration f (3) from Ref. 5 published in 2014.

The open capillary can be defined as any micro-texture on surfaces the imbibition of which results in the creation of a macroscopic liquid-air interface (see Fig. 1). This is in contrast with the case of capillary tubes, in which no macroscopic increase of liquid-air interface is observed (only the top of the liquid column is in contact with air, forming a meniscus). On the contrary, in the case of rectangular open channel or in the case of textured surfaces decorated with microscopic pillars (see Fig. 1c-e), macroscopic liquid-air interface is created as a result of the penetration of liquid into the open capillary.

This feature introduces nontrivial issues in the dynamics of capillary rise, because the geometrical shape of the large free liquid-air interface is not fixed but is dynamically determined. In fact, in the previous studies^14^,^33^,^34^where the imbibition of textured surfaces decorated with micro-pillars are reported (see Fig. 1d–e), an assumption is made for the shape of the free interface: the film is flat, i.e., the thickness of the liquid film penetrating into pillars is comparable to the pillar height (see Fig. 1d (2)). This nontrivial assumption is justified via agreement between the theory and experiments or by direct observation. This assumption is theoretically examined and justified in Ref. 41, by considering a microscopic deformation of the large liquid-air interface by pillar heads that pin the interface (see Fig. 1d (3)): the assumption can be a good approximation only when the pillar is long with a high aspect ratio (the length over radius of pillars is large) and the edge of pillar heads is sharp. In fact, when the pillars are short and the edges are rounded, the thickness of imbibed liquid film becomes smaller with the imbibition height 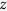, which results in a different scaling regime (see Fig. 1c): *z*  ~  *t*^1/3^^38^(this scaling law happens to be the same with that found for capillary rise into corners (see Fig. 1a-b)^36^,^37^, but the geometries are rather different). In addition, recently, for another type of open capillary found on the leg of the wharf roach *Ligia exotica*, a small animal living by the sea, yet another scaling law, *z*  ~  *t*, is found^5^(see Fig. 1f).

Given these differences found for capillary rise of various open capillaries, it would be clear that the capillary rise into rectangular open channels is nontrivial because a large free liquid-air interface is created after the imbibition of the channel. In recent studies on rectangular open channels^50^, the section of the penetrated liquid column into the channel is assumed to the same rectangle with the section of the rectangular channel. This simple geometrical approximation for the free liquid-air interface would be good when the channel is deep and the edge of the channel is sharp. However, their formula are still complex to use as simple design principles to develop microdevices. This is because they are not concerned with clarifying different scaling regimes in their formula and the range of validity of such simplified formula. In fact, to experimentally confirm their results, they used rather shallow channels, in which case the geometrical approximation is not necessarily good.

On the contrary, we here examined the case of deep channels where the approximation becomes good. As a result, we found that the complex formula for the dynamics reduces to extremely simplified forms, *z*  ~  *t*^1/2^ and *z*  ~  *e*^–ct^ (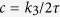), which are derived in the present study in a self-contained manner by simple scaling arguments at the level of scaling laws. In addition, we also found another scaling law *z*  ~  *t*^1/3^ for the precursor film. Practically, the *z*  ~  *t*^1/2^ dynamics is most important among the three dynamic laws for the design of mixing devices, and this important law is shown to be robust, i.e., the regimes is valid in a wide range of parameters. It is especially important for future application that the simplified laws is valid even though the channel is not significantly deep: although the laws become valid theoretically only in the limit of high aspect ratio, it is shown to be practically valid only if the aspect ratio is larger than two. This robustness of the law is practically valuable because the fabrication of rectangular channel becomes more difficult and time-consuming as the channel becomes deeper. In addition, since deeper channels require more amount of liquid, the robustness of the law is advantageous for expensive liquids. Thus, our simple results would be useful as simple design principles for the development of rectangular open channels.

Even in the well-studied case of the imbibition of textured surfaces, many issues have yet be explored. In fact, several different scaling laws have been reported^5^,^14^,^33^,^34^, for the dimensional coefficient of the relationship *z*  ~  *t*^1/2^, and the connection between the shape of the free interface and the resultant scaling law is still unclear: the crossover from the *z*  ~  *t*^1/2^ dynamics, which has been reported by several groups, to the *z*  ~  *t*^1/3^ dynamics should be clarified. In order to understand the crossover, the study of the imbibition of the rectangular open channel would be an ideal starting point, now that the wide range of applicability of the *z*  ~  *t*^1/2^ regime is established in the present paper.

In this study, we revealed that the transport of a small amount of liquid can be achieved in a controlled manner by utilizing the imbibition of the rectangular open channel and that the dynamics can be predicted almost precisely by a simple scaling law in a wide range of physical parameters. The theoretical results allow us to easily fabricate a microfluidic device for high-throughput chemical and biological reactions with no need for pumps. In principle, by tuning the geometrical design of the device under the guidance of the theoretical predictions, a small and arbitrary amount of liquid can be transported to any places on the device in a desired time, even the liquids are highly viscous as is often the case with biological liquids. In fact, we showed via a quantitative analysis that our original device can transport the BTB solution to different spots in order to start reactions at the spots simultaneously and to monitor the progress of the reactions with time. Furthermore, we showed that our another original device can mix a highly viscous liquid with another to synthesize GFP in order to monitor the progress of the expression with time via fluorescence in a quantitative manner. Our devices require only small amounts of liquids for mixing, which is especially advantageous for expensive liquids. However, since our devices are an open system, we need to care about the problems of the contamination of the system and the evaporation of liquids, which may be resolved in many practical cases. In the device for GFP, for example, it was shown by a quantitative analysis that the evaporation and contamination is not a significant problem. The open device that exploits merits of the rectangular open channel allows the transport of highly viscous liquids in a microfluidic system, which suggests that the device would be useful for outside point-of-care testing and on-site diagnostics for developing countries. In other words, the present study suggest that the scaling laws for transporting small amounts of liquids in rectangular open channels established in the present study are useful in many microfluidic applications such as droplet manipulation by flow focusing^51^, generation of concentration gradient^52^, sorting of particles^53^, and mixing^54^, in diverse fields including molecular analysis, biodefence, molecular biology and microelectronics^23^.

## Methods

### Theoretical Methods

Eq. (1): The energy 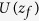 is given as 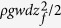 –2*g* cos θ *dz_f_* + *g* (1 – cos *θ*)*wz_f_*, (the surface terms result from 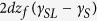 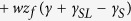 with Young’s relation 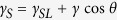). By minimizing 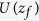 in terms of 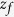, we obtain Eq. (1).

The curvature *C_f_*  ≃  2/*w*: In terms of balance between the gradients of the hydrostatic (gravitational) pressure and of Laplace’s pressure^30^, Eq. (1) is recasted in the form 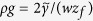. The right-hand side is interpreted as 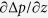 at 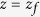 with 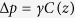 Laplace’s pressure jump and 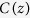 the curvature of the liquid-air interface of the liquid column at the height 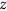, which might imply *C*(*z*)  ≃  (2/*w*) log(*z/z_0_*) 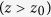 and thus *C_f_*  ≃  2/*w* for the definition 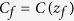.

Eq. (2): In the limit of 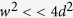, it is reasonable to assume that the flow inside the column is the Poiseuille flow of liquid sandwiched by two infinite plates separated by a distance 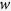 (equal to the distance of the side walls) and that the flow is induced by the above-discussed pressure gradient scaling as 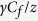. Under this assumption of infinite plate, the viscous force per unit volume is given as 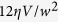 for the averaged velocity 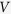 (the present approximation is in line with Darcy’s law^55^). Then, the force balance between capillarity and viscosity, 
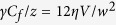
 in the limit, per unit volume, together with *V*  ≃  *dz*/*dt*, results in Eq. (2).

Eq. (4): In the same approximation for the Poiseuille flow and for the front curvature, the force balance per unit volume is expressed as 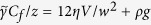, which is recast in the form, 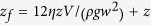. This expression is approximated as 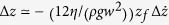 when 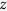 saturates nearly to the final value 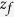 at the final stage, in which the difference 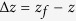 is small (with 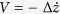). This gives Eq. (4).

Derivation of Eq. (5): The universal dynamics for the precursor film given in Eq. (5) is derived as follows. The capillary drive scaling as 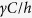 with 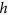 the length of precursor film is opposed by the viscous friction 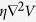 and the gravitational force 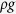 (Here, we assume the completely wetting case). The balance of the capillary drive with the gravitational force defines the curvature 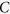 as 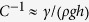. This length scale 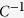 becomes smaller, indicating this to be an appropriate length for the viscous length, i.e., 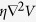 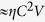. Balancing this viscous term with 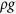 (or equivalently with 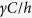), we immediately obtain the scaling law in Eq. (5) by noting 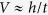. Since the film thickness cannot exceed 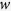 this treatment is valid only for 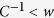, which implies that the existence of the precursor film tends to be suppressed for small height and small effective gravity.

## Experimental Methods

The open channels are fabricated on poly(methyl methacrylate) (PMMA) by a computer numerical controlled (CNC) micro-milling system (MM-100, Modia Systems) with a rigid drill bit of tip diameter 0.2 mm (NS Tool). Depending on milling conditions, the width 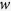 and depth 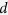 of the rectangular channel become slightly different from the designed values. In addition, the values are slightly inhomogeneous along the channel. For example, the values of 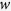 obtained by “point” measurements by a digital microscope (Keyence VHX-100) were in the range 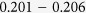 mm, not exactly equal to the tip diameter 0.2 mm.

**Figure 2:**
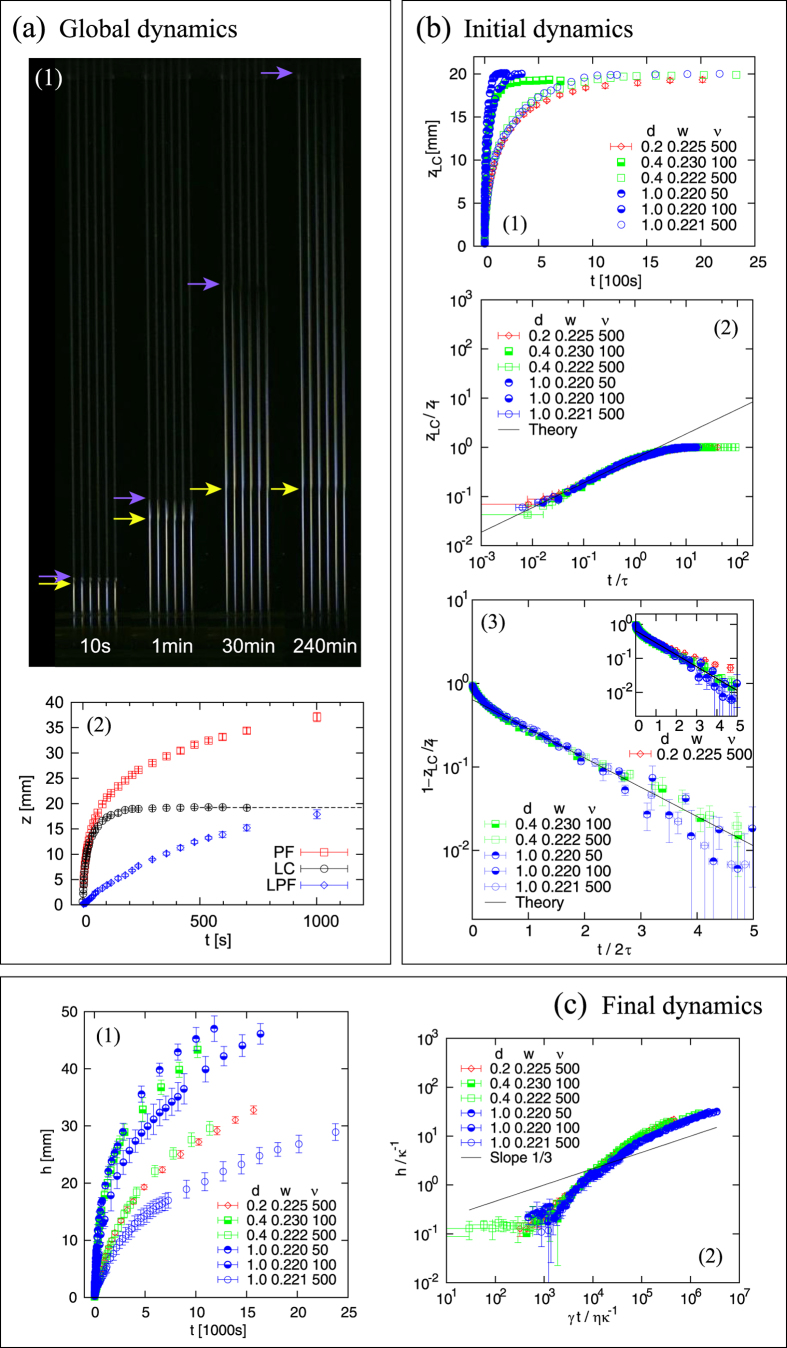
(**a**) Global dynamics. (1) Dynamics of capillary rise into open channels. Six channels with the same geometrical parameters (*d*   ≃  0.4 mm and *w*   ≃  0.2 mm) are micro-milled parallel to each other, demonstrating reproducibility of the dynamics for 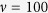 cS. The precursor film (PF) develops with time ahead of the top of the bulk liquid column (LC), below which liquid completely fills the channel; the top of LC and that of PF are indicated by arrows. (2) Front position of LC and that of PF, and the difference of the two, the length of PF (LPF), vs elapsed time 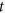 for (*d* , *w*) ≃  (0.4, 0.2) in mm and 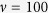 cS. The dotted horizontal line indicates the final height 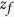. (**b**) Initial dynamics. (1) Liquid column height *z*_LC_ vs *t* for a fixed channel geometry for six sets of (*d*, *w*, *ν*). Error bars are generally small as represented by those of the data for *d*  =  0.2; Error bars are sometimes suppressed below as here for simplicity. (2) Collapse of all the data in the initial regime by the predicted theory, showing that the scaling laws obtained in the deep-channel limit are robust and holds well even for rather shallow channels. (3) Collapse of the data in the final regime by the predicted theory; The data for *d*  =  0.2 mm are removed here because they do not well satisfy the assumption of the approximate theory. The inset shows the same plot but with the data for *d*  =  0.2 mm. (**c**) Final dynamics. (1) Length of precursor film *h* vs elapsed time *t*. (2) The same plot with the axes renormalized according to Eq. (6). The straight line marked as “Slope 1/3” is a line with slope 1/3, to show that the collapsed data in the final regime approach a straight line with the same slope, as predicted by Eq. (6).

In this study, the average value of 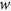 is determined from the measurement of 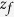. For example, we experimentally obtained 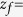 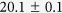 mm for 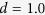 mm (“point-measured” value of 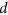 is 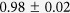 mm) both for 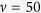 and 100 cS, where 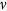 is (kinematic) viscosity. This value of 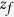 with Eq. (1) gives 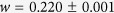 mm (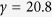 and 21.0 mN/m and 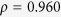 and 0.965 g/cm^3^ for 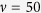 and 100 cS, respectively). This calculated value of 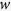 is close to the average of a limited number of “point-measured” values of 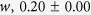 mm. In practice, partly because of the inhomogeneity of 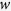 values along the channel, it is better to determine the averaged 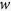 on the basis of a measured value of 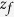 and Eq. (1), rather than to repeat “point measurements” many times, as exploited in this study. In the same spirit, in Fig. 2b (2), we used 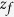 obtained from experiment, not the calculated value from the measured 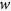 (with the microscope) on the basis of Eq. (1); when we use the latter, the collapse becomes slightly deteriorated.

The capillary rise was imaged by a USB camera (CMOS130-USB2, Fortissimo) with a lens (VS-LD20, Fortissimo). The image analysis was performed with Image J.

**Figure 3:**
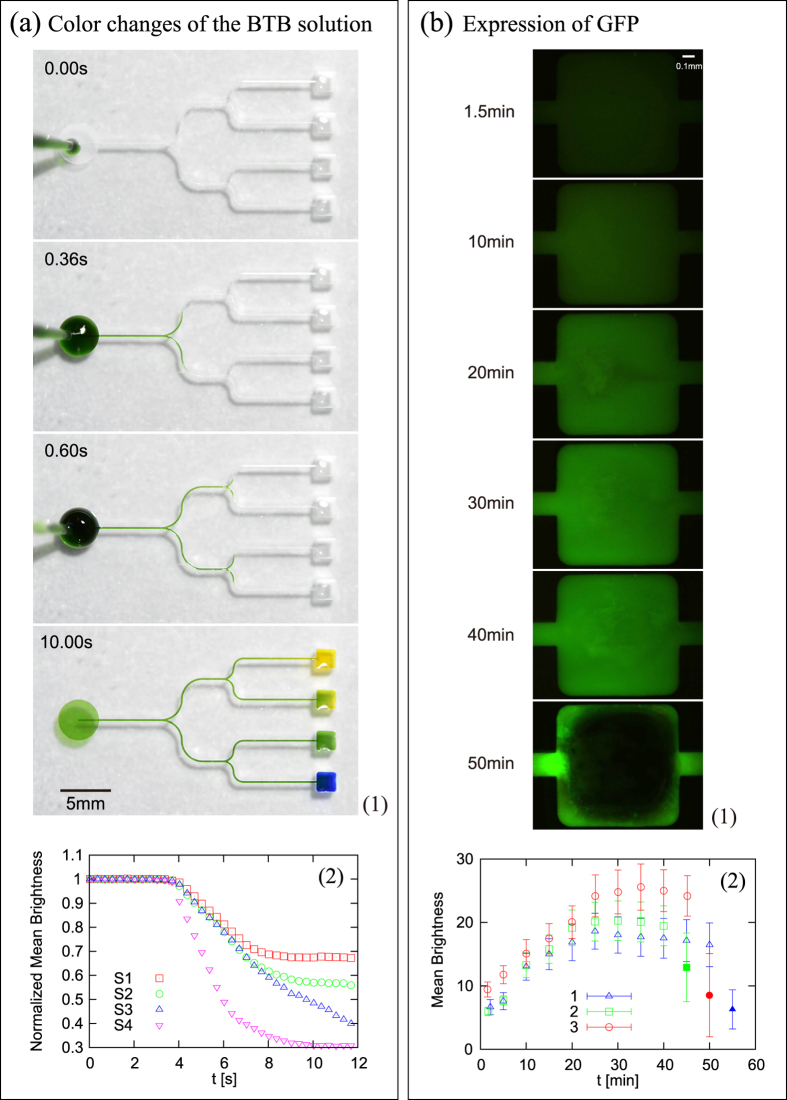
Color changes of the BTB solution. (1) A microdevice for mixing one solution with four different solutions demonstrating multiple color changes of the BTB solution depending on the pH of the solutions (all of 4.8 mm^3^) deposited in the four right rectangular spots. Fig. S1 and Movie S1 are available for this demonstration. (2) Normalized mean brightness vs time. The brightness of all the four spots in the right (S1 to S4) starts to decrease from the initial values (all normalized to one) almost simultaneously at around 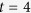 sec after the deposit of the BTB solution at the left spot at 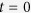, which means that the mixing and color change start to occur almost at the same time in Spots S1 to S4. (**b**) Expression of GFP. (1) Progress of the expression of GFP in a rectangular spot (of the sides 1 mm), at which a DNA solution and another (both 20 mm^3^) are mixed, on a micro-device without any pumps. Figure S2 and Movie S2 are available for this demonstration. The inhomogeneity of the color of Spot M is due to liquid flow inside the spot. The evaporation is not significant except for the bottom snapshot. (2) Brightness vs time obtained from three independent GFP experiments, showing that the device is practically useful for quantitative measurements. The data labeled 3 correspond to the experiment shown in (1). The filled symbols suggest that the liquid in Spot M is almost completely evaporated as in the bottom snapshot in (1).

A movie of the BTB experiment shown in Fig. 3a (1) was taken by a digital camera (D800E, Nikon) with a macro lens (AF-S Micro NIKKOR 60mm f/2.8G ED, Nikon); still images were procured from the movie. The pH of the solutions deposited in the right four rectangular spots were 1.2, 6.9, 10.4, and 12.8, respectively. The amount of the BTB solution deposited at the circular spot was 20 mm^3^. The normalized brightnesses in Fig. 3a (2) was calculated on the basis of an equal weight average of 256-step brightnesses for RGB colors obtained from a central part of the spot (80% of the spot area).

The details of the protein expression is given below. We used a commercial kit to express GFP (S30 T7 High-Yield Protein Expression System, Promega). The cell-free protein synthesis was performed by mixing two solutions Liquid 1 and 2, one containing DNA and the other mimicking cytoplasm, or protein expression system in bacteria (E.coli), respectively. More specifically, Liquid 1 is obtained by mixing S30 Premix Plus (15.0 mm^3^) and T7-S30 Extract for Circular DNA (13.5 mm^3^), where as Liquid 2 is obtained by plasmid DNA encoding GFP (1.5 mm^3^) and Nuclease-Free water (27.0 mm^3^).

We mixed Liquid 1 and 2 (both 20 mm^3^) by the device illustrated in Fig. S2 and detected the expression of GFP under a fluorescence microscope (IX71, Olympus) equipped with an objective lens (10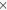) and a color CCD camera (DP80, Olympus). The exposure time and ISO are fixed to 200 ms and 800, respectively.

The brightnesses in Fig. 3b (1) and Movie S2 are multiplied by 256/140 times for RGB colors by using Image J (0-139 grade is scaled up to 0-255 grade). The brightness quantified in (2) is the equal weight average of 256-step brightnesses for RGB colors obtained from a central part of the spot (80% of the spot area).

In the GFP and BTB experiments, we performed a surface treatment to make the surfaces of the sides and bottom of channels more wettable for the aqueous solutions. The Oxygen Plasma etching for this purpose was performed by an apparatus (FA-1, Samco) under the following conditions: RF power 25W, O_3_ flow flux 10 ml/min., treating time 10 s for the BTB experiment and 30 s for the GFP experiment.

In fact, in the BTB demonstration, an advanced control of wettability is applied: only the path surfaces are made further more hydrophilic by applying hydrophilic coating liquid (WG-R1, Marusyo Sangyo), while spots for solutions with different pH are performed no surface treatments including the etching to keep the surfaces less hydrophilic to avoid leak of the solutions from the spots.

### Tips for fabricating open-channel microdevices

We can exploit Eq. (2) in designing devices with open channels. In the design of the device for the GFP expression, for example, we set the length of channel C_1_ and that of C_2_ to be the same. When the liquid deposited at S_1_ is more viscous than that at S_2_, we could tune the lengths so that simultaneous deposits at S_1_ and S_2_ lead to simultaneous arrivals at M from S_1_ and S_2_, by calculations based on Eq. (2) (if the horizontal placement of the device is possible); Otherwise, we could tune the delay time for the second deposit at S_2_ from the first deposit at S_1_ to attain the simultaneous arrivals at M, by calculations based on Eq. (2).

The recommendable values of the width 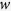 and the depth 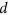 for open channels of devices fabricated with micro-milling machine can be determined as follows. The calculations for design by using the theory become more precise, when the condition 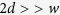 is well satisfied in principle: a “deep” channel is preferable. However, deep channels are disadvantageous in terms of fabrication and of the cost of liquids. But as demonstrated above, practically, it is sufficient if 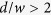 is satisfied (The constants 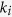 (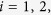 and 3) seem slightly dependent on 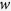 and 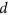). For fabrication with a micro-milling machine, a standard recommendation would be 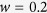 mm and 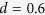 mm, as employed in our devices.

A recommended way for practical determination of the width 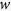 of open channels is as follows. As mentioned in Methods, depending on the milling conditions, the actual width and depth of a channel can be slightly different from desired values and, in addition, are inhomogeneous along the channel. In this respect, compared with the “point” measurement by a microscope, it is better to determine the actual effective width under the same milling conditions through Eq. (1) by measuring 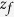 (as implied above) on a separate simpler devices as the one used in Fig. 2a (1) 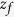 is typically of the order of centimeters so that the experiment is easy and can be performed with high precision. Note that the slight inhomogeneity in the depth of the channel does not affect the dynamics as long as 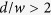 is well satisfied.

The result of the static 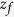 measurement with a simple device as the one used in Fig. 2a (1) can be useful to measure the viscosity 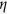 or contact angle 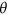 of a liquid in question through a dynamic measurement. For example, when one measures the time 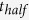 elapsed when the front of the liquid column passes through the half of the final height where still the initial dynamics applies well as mentioned above, 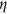 (if 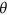 is known) or 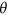 (if 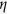 is known) is obtained from the relation, 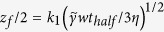. Note that the slightly less easy horizontal experiment would be advantageous in terms of precision. This is because the effect of gravity is suppressed so that the initial dynamics continues forever unless the evaporation problem comes into play.

When one wants to reduce the amounts of liquids, the width 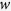 and the depth 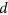 can be made smaller even to submicrons. Even in such a case, Eqs. (1) and (2) should be valid, although a possible modification may appear when the scales of the channel become close to nanoscales because of unavoidable small roughness of the surface as pointed out in the case of a closed channel^56^. In such a case, we may have to use a silicon mold made by photolithography to make a sealed channel device with PDMS gel (the seal will be preferable to reduce evaporation), which may be appropriate if the cost of liquids is significant. Note that with the seal the rectangular “approximation” becomes exact.

## Additional Information

**How to cite this article**: Tani, M. *et al.* Towards combinatorial mixing devices without any pumps by open-capillary channels: fundamentals and applications. *Sci. Rep.* **5**, 10263; doi: 10.1038/srep10263 (2015).

## Supplementary Material

Supplementary Information

Supplementary Information

Supplementary Information
