# Corrigendum: Towards combinatorial mixing devices without any pumps by open-capillary channels: fundamentals and applications

**DOI:** 10.1038/srep31017

**Published:** 2016-09-07

**Authors:** Marie Tani, Ryuji Kawano, Koki Kamiya, Ko Okumura

Scientific Reports 5: Article number: 1026310.1038/srep10263; published online: 06 23 2016; updated: 09 07 2016

In the Supplementary Information file originally published with this Article, Figures S1 and S2 were omitted. These errors have been corrected in the Supplementary Information that now accompanies the Article.

