# Supplementary material for: Towards combinatorial mixing devices without any pumps by open-capillary channels: fundamentals and applications
Source: Sci Rep. 2015 Jun 23;5:10263. doi: 10.1038/srep10263 (PMC4477624; doi:10.1038/srep10263)
Supplement: Supplementary Information [file srep10263-s1.pdf]

Supplementary Information PDF

Manuscript title

Towards combinatorial mixing devices without any pumps by open-capillary channels: fundamentals and applications

Authors

Marie Tani, Ryuji Kawano, Koki Kamiya, and Ko Okumura

Figure legends

Figure S1

Illustration of the channel pattern used in Fig. 5. The sizes are given in the unit mm.

Figure S2

Illustration of the channel pattern used in Fig. 6. The sizes are given in the unit mm.

Description of movies

Movie S1

Movie corresponding to Fig. 5. Simultaneous multiple color changes of the BTB solution are demonstrated by a micro-device that functions without any pumps.

Movie S2

Movie corresponding to Fig. 6. The expression of GFP is demonstrated by a micro-device that functions without any pumps.
